# The Role of ChatGPT in Dermatology Diagnostics

**DOI:** 10.3390/diagnostics15121529

**Published:** 2025-06-16

**Authors:** Ziad Khamaysi, Mahdi Awwad, Badea Jiryis, Naji Bathish, Jonathan Shapiro

**Affiliations:** 1 Department of Dermatology, Rambam Health Care Campus, Haifa 3109601, Israel; 2 Bruce Rappaport Faculty of Medicine, Technion Institute of Technology, Haifa 3525433, Israel; 3 Ophthalmology Unit, Tzafon Medical Center, Tiberias 1528001, Israel; 4 Dermatology Unit, Ziv Medical Center, Safed 13100, Israel; najibathish@gmail.com; 5 Maccabi Healthcare Services, Tel Aviv 6817110, Israel

**Keywords:** ChatGPT, diagnosing, dermatology

## Abstract

Artificial intelligence (AI), especially large language models (LLMs) like ChatGPT, has disrupted different medical disciplines, including dermatology. This review explores the application of ChatGPT in dermatological diagnosis, emphasizing its role in natural language processing (NLP) for clinical data interpretation, differential diagnosis assistance, and patient communication enhancement. ChatGPT can enhance a diagnostic workflow when paired with image analysis tools, such as convolutional neural networks (CNNs), by merging text and image data. While it boasts great capabilities, it still faces some issues, such as its inability to perform any direct image analyses and the risk of inaccurate suggestions. Ethical considerations, including patient data privacy and the responsibilities of the clinician, are discussed. Future perspectives include an integrated multimodal model and AI-assisted framework for diagnosis, which shall improve dermatology practice.

## 1. Introduction

Artificial intelligence (AI) has become an increasingly pivotal force in medicine, offering transformative potential across diagnostics, treatment planning, and patient care [1]. In recent years, AI applications have significantly expanded, encompassing tools like natural language processing (NLP), image analysis, and machine learning, which enable precise analysis and prediction in diverse medical fields [2]. Large language models (LLMs), like ChatGPT, now propel an important frontier of healthcare innovation. While originally designed for use as conversational agents, these are now adapted for other purposes, such as patient communication, clinical decision support, and research assistance [3].

One of the very few emerging areas where substantial promise exists for ChatGPT and other similar LLMs is dermatology diagnostics. As dermatology relies predominantly on seeing and writing descriptively accurate texts, it emerges as a distinctive context within which an AI-driven tool such as ChatGPT can glean a major benefit. This includes facilitating the interpretation and generation of medical texts and diagnostic image descriptions that could assist healthcare professionals by generating descriptions or pinpointing characteristic patterns of various skin conditions [4,5]. In addition, NLP capabilities may allow ChatGPT to automatically mine the open access medical literature to help clinicians stay up to date with complex dermatological cases and relevant guidelines [4].

Electronic medical records (EMRs) are essential for dermatology, yet much of the data remains unstructured, making analysis challenging. Natural language processing (NLP) helps streamline EMR data, enabling automated documentation, improved patient history gathering, differential diagnosis suggestions, and integration with AI image analysis. These capabilities enhance patient care, especially in teledermatology, where NLP assists in organizing patient data for remote consultations. With models like ChatGPT-4 gaining traction, understanding their role in dermatology can maximize their potential in diagnostics and clinical support [6,7,8,9].

The objectives of this review are to examine the diversified applications of ChatGPT in dermatology diagnostics, such as interpreting images, natural language processing for synthesizing and summarizing clinical knowledge, integration with machine learning, and programmatic support for dermatological research. By undertaking these dimensions, we aim to dissect the potential, limitations, and ethical perspectives that must be taken into account for responsible implementation within dermatological practice.

We will be considering ChatGPT since it represents a significant step forward in using AI in the clinical setting, particularly in dermatology, having been designed by OpenAI. It is one of the most exciting commercial products that intersects with the clinical world, especially where physicians do not have the luxury of specializing in AIs. Its NLP abilities allow it to interact with clinical data for undertaking differentiation of diagnoses, interpretations of patient symptoms, and clinical documentation. With the increasing adoption rate among dermatologists, it is imperative to carry out an exhaustive review of the social uses, benefits, and drawbacks of ChatGPT. The review will help to illustrate how ChatGPT can further improve dermatologic care while also highlighting areas where improvement is needed so that clinical applications can be used more effectively and responsibly.

### Background and Current State of AI in Dermatology

Over the last few decades, artificial intelligence (AI) has become an indispensable element in the field of dermatology diagnostics. Going from pattern recognition to deep learning systems, the evolution of AI in dermatological studies, both technologically and academically, has become more intricate [4]. Many of these early programs were designed to help clinicians recognize simple patterns in skin lesions through imaging techniques and computerized algorithms primarily to diagnose common skin conditions, such as melanoma and psoriasis [10].

Due to progress in machine learning (ML) and deep learning (DL), AI systems have become far more capable and adaptable [11]. DL models have shown to be especially useful in the analysis of skin lesions, where they learn from large sets of carefully annotated images and find patterns that are difficult for the human eye to detect [12]. For example, convolutional neural networks (CNNs) have attained an accuracy high enough to distinguish between benign and malignant lesions, sometimes performing at a level comparable to that of dermatologists [13,14]. However, traditional AI models have limitations. These include problems of generalizability across populations and lighting conditions and reliance on large, diverse datasets that are often difficult to collect and annotate within dermatology [15,16].

The development of AI has ushered in the era of large language models (LLMs), including OpenAI’s ChatGPT, which do indeed mark a change in utility for AI in fields of medicine like dermatology. While LLMs began with the purposes of natural language processing, they have become advanced enough to process and generate text in a manner similar to that of humans [17]. Unlike conventional ML or DL models, which mainly focus on image analysis, LLMs like ChatGPT can analyze text inputs for clinical decision support and facilitate patient–physician communication by explaining diagnostic reasoning or treatment options in lay language [18]. Such versatility makes LLMs an exciting addition to dermatology, especially in areas where image-based AI tools fall short, like patient-specific concerns or contextualizing image findings with patient histories [19].

As an AI algorithm, ChatGPT can contribute to diagnostic workflows beyond the limits of image-only models through analysis of substantial amounts of general medical knowledge such as addressing patient concerns, providing proofs detailing the diagnosis through evidence-based insights [20], and even an interpretation of complicated skin conditions through detailed descriptions given by the patient. For instance, ChatGPT can offer believable differential diagnoses or suggest follow-ups, especially advantageous in a primary-care setting [21], where dermatologists are not readily accessible. However, while this area may be promising, it is imperative to approach the integration of LLMs such as ChatGPT trained on dermatology datasets cautiously, as they are not free from certain limitations, for example, precision-dependent cases or situations requiring critical diagnostic accuracy [22].

## 2. Image Analysis Using ChatGPT and Its Integration with Diagnostic Tools

In dermatology, image analysis plays a pivotal role in diagnostics, for clinical images, dermoscopic images, histology images, and confocal microscopy images, with the use of instruments like dermoscopy and clinical imaging giving us high-resolution views that not only help with early detection but also facilitate accurate diagnosis of conditions like melanoma and other skin problems [23]. Dermatoscopic images play, in particular, a key role in enabling dermatologists to detect fine changes occurring within skin lesions since such structures are not readily visible to the naked eye. All of that, in turn, practically demonstrates drastic increases in diagnostic accuracy [24]. ChatGPT, being a large language model, is notable for interpreting prompts and formulating self-consistent text descriptions. Thus, processing detailed image-related descriptions for clinicians can assist ChatGPT in providing relevant information for potential diagnostic implications or possibly suggest areas for further examination [25]. Such capability enables dermatologists to use ChatGPT as an auxiliary tool for the generation or refinement of descriptions, notably in cases where immediate computer vision assessment is unavailable.

With descriptive assistance, ChatGPT may help dermatologists generate detailed and relevant descriptions emphasizing clinical signs and, potentially, minimize human errors while improving diagnostic accuracy [26]. ChatGPT can, therefore, serve as an assistance for the clinician in potentially rephrasing observations or suggesting an alternative diagnostic wording to elaborate an uncertain lesion description in a complex case; by adding the input of an expert, increased reliability and diagnosis were achievable [27].

Incorporating computer vision (CV) neural networks and deep machine learning frameworks into ChatGPT might enhance the existing setup of diagnostic workflows in dermatology [28]. The output of CNNs, trained on extensive datasets and influential in the constructive identification of features of skin lesions, could do much better with the input of ChatGPT for its natural processing of language in preparation for interpretive summaries or comments that could provide human interpreters with a little help [28,29]. Thereby, they adopt a symbiotic approach that involves portable knowledge pairing that combines the strengths of both systems, whereby computer vision interrogates the feature identifications and associated descriptions to relate this information to a clinically relevant narrative.

Previous GPT models more exclusively input text data and interpreted visual data with detailed descriptions. Long outputs were often not described properly or lost something vital, especially in areas like dermatology, where visual subtleties have always been in question [30]. With GPT-4’s multimodal capabilities, the limitation has been somewhat circumvented as the model can directly analyze an image together with text. But GPT-4 is still behind the achievements of specialized computer vision models for medical imaging and lacks the ability to cross-check its interpretations. This means that it could be a very handy adjunct but is not useful as an independent diagnostic tool.

**Figure 1:**
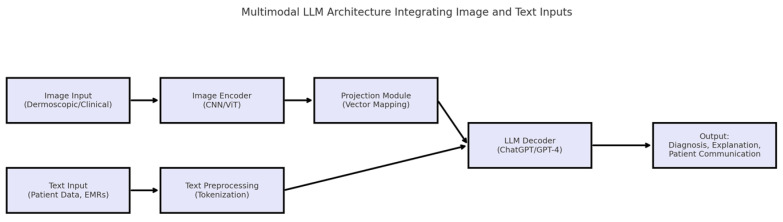
Illustration of a multimodal large language model (LLM) system integrating both image and text data. The image pathway is encoded via convolutional neural networks (CNNs) or vision transformers (ViTs), while text inputs (e.g., patient history) are tokenized and processed by a large language model (e.g., GPT-4). A projection module aligns visual embeddings with the text domain, enabling the model to generate comprehensive outputs such as diagnostic suggestions, patient education, and clinical explanations.

The concomitant improvement of new developments in dermatologic diagnostic technologies may lie with the combination of off-the-shelf large language models, like ChatGPT, and next-generation computer vision technologies, thus developing a joint exterior approach, wherein LLMs process visual data properly with clinic parameters of such nature [22]. A conceptual overview of such a multimodal system is illustrated in Figure 1.

The evolving LLMs, in the process of interacting more directly with visual input, could provide predictive insights along with image analysis, streamlining diagnostics and maximizing early detection of dermatologic conditions. Such advances could lead to systems where LLMs like ChatGPT continuously refine image interpretations made by CNNs, culminating in a hybrid diagnostic model incorporating textual and visual analytics in an integrated approach serving clinical support [10,12].

## 3. Natural Language Processing (NLP) Capabilities of ChatGPT in Dermatology

NLP has already become a disruptive technology in the healthcare domain, mainly in diagnostics and patient management. NLP can analyze unstructured data in EHRs—patient notes, clinical histories, and diagnostics reports—which helps healthcare providers gain impressions that facilitate better and more accurate decisions in diagnosing and planning. In pathology, NLP can help to extract relevant data from the patient history and allow quick analysis of text-based information, enabling diagnostic workflows while easing the workload of a clinician [6,7,8,9].

Another shining example of efficient natural language processing developed by OpenAI in the dermatological field is ChatGPT. The pertinent information that really could guide a clinician’s diagnostic decision has now been extracted, analyzed, and presented on a platter by ChatGPT, using the patient anamnesis and clinical notes. To put it plainly, while scrutinizing patient medical histories, ChatGPT can present dermatologic symptoms along the lines of pruritus, erythema, and lesions, in this way allowing better initial assessments. In addition, ChatGPT is able to parse out relevant dermatologic symptoms among free-text inputs, allowing clinicians a more efficient means of garnering information needed in diagnostic metrics, which leads to smoothening the initial triage process in a wide range of clinical and telemedicine settings [6,9,30,31].

Teledermatology, telemedicine for the diagnosis of skin conditions, has gained ground in recent years, ever since newly found demands for remote healthcare solutions arose. ChatGPT’s new-wave NLP capabilities constitute a boost to documentation tools, wherein automated summaries of patient interactions and symptom reporting are significant aiding features. These features permit dermatologists to concentrate on the case, ensuring that clinically necessary medical records are accurate and comprehensive. Further, it helps communication by making sure the responses generated sufficiently cover the questions, clarifications of instructions, and laying down the treatment plan in a manner a patient can understand, especially when referring to a patient seeking dermatoscopy through virtual modalities [8,9,30,32,33,34].

Recent studies have begun with a glance at the applicability of ChatGPT and its equivalent natural language processing models in dermatology. In one of the studies analyzed, ChatGPT was competent enough to extract disease-specific terminology from dermatological case notes in support of improving the diagnostic work of clinicians [6,7,26]. Another study suggested that ChatGPT and various NLP approaches may further compress and summarize patient records, as well as create documentation errors in other medical methods [26,35,36]. Based on comparisons with traditional dermatological diagnostic means, some studies used OpenAI’s ChatGPT and other models of NLP to help non-specialist providers identify common skin diseases, which would allow these patients more access to dermatological services in under-served areas [9,37].

Like any other natural language processing technologies used in the healthcare system, ChatGPT in dermatology presents significant ethical concerns with regard to patient data, confidentiality, and privacy. The model must conform to the legality of patient confidentiality, meaning all data protection standards must be observed to guarantee patient secrecy, such as compliance with HIPAA in the United States. Further, there lies an ethical dilemma pertaining to the accuracy of NLP: in precise words and interpretation, this may lead to improper diagnoses. In a way, therefore, it presents risk in the automated processing of clinical data in dermatology, and hence appropriate data governance mechanisms and oversight from clinicians are necessary to mitigate risks associated with automated data processing [38,39,40].

## 4. Machine Learning and Programming Applications in Dermatology Diagnostics

ChatGPT is a powerful tool that researchers use to create and refine ML algorithms [41] oriented toward dermatology diagnostics. These revolve around feature selection, model optimization, and data preprocessing strategies tailored to meet clinical requirements and fit within the data structure [42]. For example, it may stipulate ways to improve performance in convolutional neural networks (CNNs) while analyzing dermatological images, possibly by data augmentation, thereby reducing overfitting [28,43,44]. ChatGPT greatly aids dermatology researchers in developing, optimizing, and debugging their programming skills, so they can perform programming activities more quickly [9,45]. For example, it can help develop Python scripts in training ML models on dermatological image datasets, creating pipelines for data preprocessing on skin lesion image datasets, or solving case problems of integrating libraries like TensorFlow (version 2.19.0) or PyTorch (v2.7.0) into existing workflows. ChatGPT can also provide explanations for coding errors or suggest alternative methods to improve computational efficiency [46,47].

## 5. Summary of the Literature Findings on Machine Learning and Programming Applications in Dermatology Diagnostics

Recent advances in artificial intelligence widen its applications in dermatology, and ChatGPT emerges as a new tool to aid in diagnosis. This review presents findings from the studies about ChatGPT’s utility in dermatological diagnosis, being compared with traditional AI models, and outlines its reception by dermatologists and patients.

ChatGPT has been evaluated for its potential in dermatology diagnostics in a few studies. For example, [Ferreira et al., 2023] found that ChatGPT offers competent evidence of recognizing some symptoms of conditions like eczema and acne, showing an 88% agreement with expert dermatologists [37]. In the same way, [Lam Hoai & Simonart, 2023; Shapiro et al., 2024] evaluated its attempts at giving treatment advice, reporting a moderate to high degree of accuracy in conformity with current clinical guidelines [6,7].

ChatGPT has recently been compared with traditional AI models in dermatology, bringing various strengths and performance to light. One study comparing ChatGPT and Claude 3 Opus examined their respective capabilities with both malignancies and benign birthmarks of 100 dermoscopic images. ChatGPT was more correct in classifying the top three diagnoses than an alternative technology, being slightly superior at 78% to 76%, while Claude 3 Opus, having higher sensitivity and specificity, was more competent than ChatGPT in malignancy discrimination. ChatGPT, with a better breadth of knowledge and contextual considerations, sometimes failed to classify benign conditions as being malignant, a concern that underlines the necessity for better fine-tuning before clinical use [48].

One of the major areas of study that is focused chiefly on is the user experience of ChatGPT. Based on the findings of Goktas and Grzybowski, ChatGPT was found to be conversationally useful in supporting patient education and decision-making [45,49]. In the meantime, [Alanezi, 2024] validated that the patients had high satisfaction with the use of ChatGPT for preliminary consultations, adding that accessibility and a non-threatening interface were the main strengths [50].

In a recent review to assess the performance of ChatGPT for a multitude of dermatological conditions, it was shown to have varying degrees of good and poor accuracy. According to studies, ChatGPT has been cited with high accuracy in issues such as psoriasis and eczema, yet users complained of poor performance with more complex instances of, say, cutaneous neoplasms, otherwise complicated by the fact that some of these neoplasms require nuance for proper identification. For example, ChatGPT fared better in identifying benign lesions by description but had serious trouble correctly diagnosing malignant lesions compared to image-based AI models.

There was a large variance in the performance, necessitating improvement in the size of the dataset or adjustment to the models of improvement so that the complexity and variability of dermatological manifestations may be dealt with [45].

The reliability of ChatGPT in generating accurate differential diagnoses remains a topic of ongoing debate. Some studies highlight its ability to provide consistent and reasonable diagnostic suggestions for common dermatological conditions, demonstrating its potential as a supportive tool in clinical settings [7,9,45]. However, other research highlights occasional inaccuracies, particularly in cases involving rare or atypical presentations [48,49,51]. These discrepancies underline that while ChatGPT can complement clinical efforts by offering differential diagnoses, it should not be relied upon as a standalone diagnostic tool. Experts consistently emphasize its role as an adjunct to enhance, rather than replace, professional judgment.

To date, several studies on the uses of Generative Pre-trained Transformers (GPTs) in dermatology have explored various domains in diagnostics, therapy recommendations, and patient education. More precisely, Table 1 presents a summary of 18 key studies that picture the variety of GPT applications.

The mainstay across several studies (10) illustrates the capacity of GPTs in dermatologic diagnosis, allowing treatment through text, multimodal, or image-based systems. For instance, GPT-4 impressed with extraordinary diagnostic accuracy across several dermatologic conditions in both text and multimodal examples, but does not yet show a satisfactory level of accuracy in image-based diagnosis, as discussed by Pillai et al. [52]. In comparative studies, such as Liu et al., the strength of GPTs was examined against that of other AI models, showing exceptional areas as well as those needing refinement in melanoma diagnosis [18].

Less research has focused on management and treatment recommendations, but a study by Iqbal et al. indicated that GPT-4 may offer parallel second opinions for dermatology treatments that earned wide approval among dermatologists [53]. Yet it has trouble with more difficult cases, which opens up room for enhancement.

As for educational and examination performance, GPT-4 is notedly more effective than its predecessors, attaining accuracies from 75 to 93% in various dermatological examinations (Elias et al.; Passby et al.) [54,55]. These works show promise in being able to apply GPT-4 to vetting an adjunct to medical education; however, the shortcomings involving high-difficulty questions indicate a need for clinical assessment on its behalf.

Finally, studies on NLP applications have indicated GPT’s participation in conducting an analysis of unstructured patient data and building materials of patient education, to cite examples like Shapiro et al., where GPT-4 provided high accuracy amid psoriasis patient records [6], and Lambert et al., where it generated accessible patient education materials tailored to different reading levels [56].

In general, while most studies center on its diagnostic applications, the growing exploration of GPT in treatment and education reflects the growing interest in its multifaceted potential. However, the consideration of further research into the integration of GPT into dermatology is needed due to various challenges such as ethical concerns, limitations in dealing with complex cases, and variable performance. An overview of the published studies on ChatGPT and other LLMs in dermatology is summarized in Table 2.

**Table 1:** Categorization of studies on GPT in dermatology.

Type	Focus Area	Paper Title/Details	Authors	Year	Summary of Findings	Version of GPT	Image Dataset Source	Diagnosis Compared to	Study Type	Diagnosis Made Based on Images Alone or Metadata	Image no.	Types of Images
Diagnostics	Image Processing	Evaluating the Diagnostic and Treatment Recommendation Capabilities of GPT-4 Vision in Dermatology [52]	Pillai et al.	2024	GPT-4V demonstrated strong diagnostic accuracy for dermatological conditions, especially in text-based scenarios, with 89% accuracy in both text and multimodal setups. However, its image-based diagnosis showed lower performance, highlighting the need for further model development.	GPT-4.0	Publicly available sources: dermnet.nz and dermatlas.org.	Two board-certified dermatologists	N/A	A combination of both images and metadata	54 images.	Images depicting 9 common dermatological conditions, showcasing classic manifestations of these conditions.
		Argentine dermatology and ChatGPT: infrequent use and intermediate stance [57]	Ko et al.	2024	A survey of 257 Argentine dermatologists showed 83.7% were familiar with ChatGPT, but 65.4% had never used it. While 74.9% expressed interest in future use, only 5.4% used it frequently. Most were ‘early majority’ adopters.	N/A	N/A	N/A	Prospective	N/A	N/A	N/A
		Claude 3 Opus and ChatGPT With GPT-4 in Dermoscopic Image Analysis for Melanoma Diagnosis: Comparative Performance Analysis [48]	Liu et al.	2024	This study compared the diagnostic performance of Claude 3 Opus and ChatGPT for melanoma detection, finding no significant difference in primary diagnosis accuracy but superior malignancy discrimination by Claude 3 Opus. Both models showed potential, but their limitations highlight the need for further development in AI-driven dermatology tools.	GPT-4.0	The International Skin Imaging Collaboration (ISIC) archive.	N/A	N/A	Image	100	Dermoscopic images of melanocytic lesions.
		ChatGPT versus clinician: challenging the diagnostic capabilities of artificial intelligence in dermatology [58]	Stoneham et al.	2024	ChatGPT correctly diagnosed 56% of cases with expert data and 39% with non-specialist data, lower than dermatologists (83%). It always provided a differential diagnosis but did not significantly improve diagnostic accuracy in primary or secondary care.	GPT-4.0	N/A	The diagnosis made by ChatGPT was compared to those made by dermatologists (experts) and nonspecialists.ective	Retrospective	Metadata	N/A	N/A
NLP		A Qualitative Analysis of Provider Notes of Atopic Dermatitis-Related Visits Using Natural Language Processing Methods [8]	Pierce et al.	2021	This study analyzed provider notes for 133,025 patients with atopic dermatitis (AD), revealing a focus on symptoms (primarily itch) and treatment, but limited documentation of AD’s impact on patients’ work or lifestyle. The findings highlight a care gap that requires further investigation.	N/A	N/A	N/A	Retrospective	N/A	N/A	N/A
		Application of a natural language processing artificial intelligence tool in psoriasis: A cross-sectional comparative study on identifying affected areas in patients’ data [6]	Shapiro et al.	2024	ChatGPT-4 accurately analyzed unstructured EMR data from psoriasis patients, identifying affected body areas with 92.8% accuracy. It demonstrated high performance in detecting nail and joint involvement, though errors were more common in complex cases.	GPT-4.0	The study does not involve images; it uses unstructured text data from EMRs.	Senior dermatologist	Retrospective	Metadata	N/A	N/A
		Comparing Meta-Analyses with ChatGPT in the Evaluation of the Effectiveness and Tolerance of Systemic Therapies in Moderate-to-Severe Plaque Psoriasis [7]	Lam Hoai et al.	2023	ChatGPT-4 accurately analyzed psoriasis patient data, identifying affected areas with 92.8% accuracy. It performed well in detecting nail and joint involvement, though errors occurred in complex cases.	N/A	The study does not mention the use of an image dataset; it focuses on evaluating textual data and conclusions from meta-analyses.	Experts	Retrospective	The study did not involve image-based diagnosis; it focused on evaluating textual conclusions from meta-analyses and ChatGPT outputs	N/A	N/A
Patient Interaction	Use of ChatGPT for Query Handling	Trends in Accuracy and Appropriateness of Alopecia Areata Information Obtained from a Popular Online Large Language Model, ChatGPT [59]	O’Hagan et al.	2023	ChatGPT 4.0 demonstrated higher accuracy (4.53/5) than ChatGPT 3.5 (4.29/5) in addressing patient questions about alopecia areata. Responses were rated highly appropriate for general information and moderately suitable for EHR drafts, indicating potential for patient education and clinical use.	N/A	N/A	N/A	N/A	N/A	N/A	N/A
		Comparing the quality of ChatGPT- and physician-generated responses to patients’ dermatology questions in the electronic medical record [9]	Reynolds et al.	2024	This study was evaluating responses to patient questions, physician-generated responses were preferred over ChatGPT’s, especially for readability and empathy. However, ChatGPT was seen as useful for drafting initial responses and providing educational information.	GPT-3.5	N/A	The diagnosis was compared to responses from dermatology physicians, as well as nonphysicians (blinded reviewers).	Retrospective	N/A	N/A	N/A
	Generates learning materials	Assessing the Application of Large Language Models in Generating Dermatologic Patient Education Materials According to Reading Level: Qualitative Study [56]	Lambert et al.	2024	LLMs like GPT-4 generate dermatologic patient education materials (PEMs) at specified reading levels, with GPT-4 performing best at the fifth-grade level for both common and rare conditions. PEMs produced by LLMs are generally accurate, easy to read, and understandable for patients, with variable results at the seventh-grade level.	ChatGPT-3.5, GPT-4.0.	N/A	The diagnosis was compared to 2 blinded dermatology resident trainees.	N/A	N/A	N/A	N/A
Others	Performing exams	ChatGPT-3.5 and ChatGPT-4 dermatological knowledge level based on the Specialty Certificate Examination in Dermatology [60]	Lewandowski et al.	2024	ChatGPT-4 outperformed ChatGPT-3.5 in dermatology exams, achieving 80–93% accuracy in English and 70–84% in Polish. While effective in clinical decision support, it struggles with high-difficulty questions. Recommended for aiding but not replacing physicians.	ChatGPT-3.5, GPT-4.0.	N/A	The study compared the performance of ChatGPT to that of a dermatologist with 25 years of experience, who reviewed the questions for compliance with current knowledge.	Retrospective	N/A	N/A	N/A
		Performance of ChatGPT on Specialty Certificate Examination in Dermatology multiple-choice questions [55]	Passby et al.	2024	ChatGPT-4 scored 90% on 84 multiple-choice dermatology questions, outperforming ChatGPT-3.5 (63%). This highlights AI’s potential in clinical decision-making, with caution regarding complex cases and patient safety.	ChatGPT-3.5 and ChatGPT-4.	N/A	N/A	N/A	N/A	N/A	N/A
		OpenAI’s GPT-4 performs to a high degree on board-style dermatology questions [54]	Elias et al.	2024	GPT-4 achieved 75% accuracy on dermatology board-style questions, showing potential as an educational tool but requiring improvements in response depth and completeness for unsupervised learning. Its performance was consistent across subspecialties and question difficulty.	GPT-4.0	N/A	The diagnosis was compared to the correct answers evaluated by two physicians.	Cross-sectional study	N/A	N/A	N/A
		Pediatric dermatologists versus AI bots: Evaluating the medical knowledge and diagnostic capabilities of ChatGPT [61]	Huang et al.	2024	This study compares OpenAI’s ChatGPT (versions 3.5 and 4.0) to pediatric dermatologists in answering multiple-choice and case-based questions. Results show that while human clinicians outperformed both AI versions, ChatGPT-4.0 performed comparably in some areas, highlighting AI’s potential with clinician oversight.	GPT-3.5 and GPT-4.0	The image dataset was not used. For cases with accompanying images, only text descriptions were included	The diagnosis was compared to pediatric dermatologists	prospective	text descriptions alone	N/A	N/A
	management	An evaluation of ChatGPT compared with dermatological surgeons’ choices of reconstruction for surgical defects after Mohs surgery [62]	Cuellar-Barboza et al.	2024	This study found that while ChatGPT-4 showed slight concordance with dermatologists in reconstructive decision-making for skin cancer surgery, the agreement was lower than that between dermatologists themselves. The findings highlight the variability in AI-driven medical decisions and the importance of certified expertise.	ChatGPT-4.0	N/A	The diagnosis was compared to dermatological surgeons’ choices.	Retrospective	N/A	N/A	N/A
		Evaluation of ChatGPT’s acne advice [63]	Li et al.	2024	This study assessed ChatGPT’s responses to acne-related queries, evaluating accuracy, completeness, and relevance. Dermatologists rated the responses as satisfactory, limited, or problematic, revealing variable quality in the answers.	ChatGPT-3.5	N/A	The diagnosis was compared to two board-certified dermatologists	Retrospective	N/A	N/A	N/A
Machine Learning	Diagnostics	Pre-trained multimodal large language model enhances dermatological diagnosis using SkinGPT-4 [64]	Zhou et al.	2024	SkinGPT-4 is an interactive dermatology diagnostic system that combines a vision transformer and Llama-2-13b-chat, trained on 52,929 skin disease images. It autonomously diagnoses skin conditions, analyzes characteristics, and offers treatment recommendations based on real-life evaluations with dermatologists.							
	Treatment	Can large language models provide secondary reliable opinion on treatment options for dermatological diseases? [53]	Iqbal et al.	2024	Proven potential to provide accurate second opinions on dermatological medication recommendations (98.87% approval rate by dermatologists). However, limitations include occasional coding inaccuracies and incomplete data, suggesting the need for domain-specific knowledge integration.							
	Exams	Assessing large language models’ accuracy in providing patient support for choroidal melanoma [65]	Anguita et al.	2024	ChatGPT provided the most accurate answers (92%) for medical advice questions about choroidal melanoma compared to Bing AI and DocsGPT. However, inconsistencies highlight the need for fine-tuning and oversight before clinical use.					Assessing large language models’ accuracy in providing patient support for choroidal melanoma [65]	Anguita et al.	2024
		Performance of Three Large Language Models on Dermatology Board Examinations [66]	Mirza et al.	2024	GPT-4 outperformed GPT-3.5 and Google Bard in dermatology board-style questions, achieving 81.7% accuracy and passing CORE and APPLIED exams. Challenges included difficulty with higher-order and complex questions.					Performance of Three Large Language Models on Dermatology Board Examinations [66]	Mirza et al.	2024
[53,64] Reviews	Review—diagnostics	Assessing the Impact of ChatGPT in Dermatology: A Comprehensive Rapid Review. [67]	Goktas et al.	2024	ChatGPT shows promise in patient education and teledermatology but faces challenges in diagnosing complex cases and raises ethical concerns regarding data privacy and algorithmic bias. Future research should focus on improving its diagnostic accuracy and addressing these issues.							
	General review on the potential	ChatGPT and dermatology [68]	D’AGOSTINO et al.	2024	This review explores the potential applications of ChatGPT in dermatology, highlighting its role in clinical practice and patient support. It emphasizes the synergy between AI and dermatology, driving innovation in healthcare delivery.							
		Potential applications of ChatGPT in dermatology [69]	Kluger	2023	Supports clinical decision-making and treatment planning with high accuracy for common conditions. Facilitates patient education, simplifies medical writing, and integrates into teledermatology platforms for consultations and triage. Limitations include challenges in multilingual settings, image interpretation, and ethical concerns.							
		Ethical considerations for artificial intelligence in dermatology: a scoping review [70]	Gordon et al.	2024	AI applications span mobile apps for skin cancer detection, clinical image analysis, and large language models for diagnostic queries. Ethical concerns include biases, misdiagnosis risks, data privacy, and exacerbation of health disparities in teledermatology. Benefits include improved access, decision-making, and efficiency in clinical practice, but safeguards are necessary for ethical use.							
	Analyzing potential	ChatGPT for healthcare providers and patients: Practical implications within dermatology [71]	Jin et al.	2023	Identified five domains of use in dermatology, including automating administrative tasks, enhancing patient education, supporting medical education, aiding clinical research, and improving health literacy. Ethical challenges include risks of “artificial hallucinations,” biases, and outdated datasets, necessitating systematic validation and usage guidelines.							
		The Arrival of Artificial Intelligence Large Language Models and Vision-Language Models: A Potential to Possible Change in the Paradigm of Healthcare Delivery in Dermatology [19]	Gupta et al.	2024	The study explores the potential of large language models (LLMs) and vision-language models (VLMs) in dermatology, addressing how AI can improve patient care amidst challenges like workload and staffing shortages. AI technologies, such as ChatGPT and Google Bard, could transform dermatology by integrating text and image inputs.							

## 6. Limitations of Using Large Language Models (LLMs) Like ChatGPT in Dermatology Diagnostics

Though LLMs like ChatGPT have shown possibilities in aiding dermatology diagnostics, they still hold some considerable shortcomings. The inability to interpret dermatoscopic or clinical photographs directly is a major limitation; this is fundamentally central to the diagnosis of most skin conditions. Therefore, their incapacity to make independent diagnoses is largely due to their dependence on other tools or their joint incorporation with image-processing systems [72,73]. Nonetheless, the introduction of GPT-4 has partly resolved this limitation. In this regard, GPT-4, by combining with vision humanoid models or external APIs, has the possibility to process and analyze pictures to some degree, rendering dermatological practice more relevant by joining both text and visual diagnostic workflows [74].

Despite these advancements, the risk of “hallucination”—generating incorrect or misleading suggestions—remains a concern, particularly in high-stakes clinical scenarios [75]. Furthermore, dermatologic conditions with similar visual presentations often require nuanced differential diagnosis, a task that AI-based imaging tools may not reliably perform without contextual clinical information [48,58]. Furthermore, these models still require integration with domain-specific knowledge bases to ensure relevance and accuracy, as their general training data often lack the granularity necessary for specialized medical contexts [76,77].

It is worth mentioning that uploading images to GPT may result in some ethical consequences. Before sharing images, patient permission must be obtained, taking dignity and confidentiality into account. Temporary chat mode—the automatic deletion of the conversation and any uploaded data when the session closes—is preferable in terms of potential risks. This would further avoid abuse and guarantee better privacy protection. Additionally, one such capacity in allowing this temporary and single-session interaction gives evidence of a huge advantage to keeping ethics in place when such AI technologies are used in healthcare [70,78,79].

## 7. Future Perspectives

The future of integrating LLMs like ChatGPT into dermatology looks bright, with several anticipated developments listed below:

- Better Integration with Diagnostic Tools:

The combination of ChatGPT with dermatoscopes, smartphone applications, and other diagnostic technologies has the prospective of creating an improved workflow of clinicians, facilitating rapid data interpretation and prompt decision-making. For instance, linking ChatGPT with a smartphone app specially for clinical use would allow for real-time, dialogue-based analyses of patient-reported symptoms in conjunction with visual data from dermatoscopic images.

- Multimodal Models:

Future AI systems combining text and image analysis stand to provide a synthesis of a holistic diagnostic approach. These multimodal models could better connect the language-functioning domain of ChatGPT with various imaging technologies in improving diagnostic accuracy for those conditions requiring visual assessment, such as melanomas and psoriasis.

- Personalized Treatment Recommendations:

LLMs could be personalized to incorporate patient-specific data like genetic profiles, medical history, and lifestyle preferences into treatment recommendations. This will enable dermatologists to provide more targeted accompaniment to them.

- AI-Assisted Diagnostic Frameworks:

The development of frameworks involving models like ChatGPT could reform the practice of dermatology following AI-assisted consultations and a clinical support system for diagnosis that would enhance clinical efficiency and precision.

- Future Research Directions:

Longitudinal studies assessing the long-term reliability and outcomes of these technologies are needed for the promise of AI in dermatology to be fully realized. These can include real-world implementation projects and clinical trials aimed at their exploration of efficiency and scalability, ensuring AI models produce unbiased functioning across different populations and settings.

## 8. Conclusions

The integration of ChatGPT and similar LLMs in dermatology is a major step forward toward assistance in diagnosis and clinical support. While the model offers significant advantages, especially in terms of natural language processing and image analysis, it is fraught with limitations that could affect the diagnostic interpretation of visual laboratory tests and assignments. ChatGPT can also serve as a very helpful assistant for dermatologists to speed up the processing of patient information and differential diagnosis particularly in teledermatology and remote care contexts. Nevertheless, we argue for further research and development to optimize such instruments. A more accurate, self-informing tool would still command the highest approach because reputation is at stake as far as these case studies are concerned, alongside all the possible benefits and challenges like data privacy and clinical liability. Owing to the continued evolution of AI technology, the future of dermatology will involve a more seamless integration of AI diagnostic tools for always better efficiencies, accuracy, and equitable patient care improvements.

## Figures and Tables

**Table 2:** Volume of articles on ChatGPT vs. other LLMs in dermatology diagnostics.

Model	Volume of Articles	Applications in Dermatology
ChatGPT (OpenAI)	High (50+ articles)	Used for diagnostic support, clinical note analysis, and patient interaction. Achieves 88% accuracy in handling common queries.
Google Bard (PaLM)	Low (2–5 articles)	No specific dermatology focus found; primarily applied to general conversational AI and integration with Google systems.
Claude by Anthropic	Low (1–2 articles)	Limited mentions in healthcare; prioritized for safe, ethical applications in sensitive tasks.
Microsoft Copilot (Powered by GPT-4)	Moderate (5–10 articles)	Integrated in teledermatology workflows via Office Suite for clinical reporting and diagnostic data organization
LLaMA (Meta)	Low (<5 articles)	Primarily used in researchno specific dermatology-related applications identified.
Mistral	None Found	No known dermatology-related applications.
Cohere	None Found	Primarily enterprise knowledge management; no dermatology-specific use cases identified.
Amazon Bedrock	None Found	Focus on general enterprise flexibility; no dermatol no dermatology-specific use cases identified.
xAI (Grok)	None found	Still emerging; no dermatology-specific use cases identified.
IBM Watson Assistant	Low (1–2 articles)	Some use cases in patient engagement and healthcare support, but minimal focus on dermatology.
Jasper AI	None Found	No known applications in dermatology; focused on content creation.
Character.ai	None Found	Used for entertainment; no healthcare or dermatology use cases.
DeepMind Gemini	Emerging	Promising capabilities in diagnostics, but still in development with no dermatology applications yet.
Perplexity.ai	None Found	Focused on information retrieval with no dermatology-specific applications.

Table 2 displays that among the evaluated LMMs, very few have published studies on their use in dermatology. In this regard, ChatGPT had the most number of articles published: over 50 studies on various applications, including diagnostic support, clinical note analysis, and patient interaction, with an 88% accuracy rating for handling common queries. Microsoft Copilot is powered by GPT-4 with 5–10 articles published, mainly about its integration into teledermatology workflows, clinical reporting, and organizing diagnostic data. Google Bard (PaLM) has 2–5 published studies, and Claude by Anthropic has 1–2 published studies; however, these two do not specifically address dermatology, as they have been more generally applied to broader conversational AI tasks. Other models with limited publication on dermatology include IBM Watson Assistant, LLaMA, and emerging models such as DeepMind Gemini. Many models, such as Mistral, Cohere, and Amazon Bedrock, have no known applications in the field of dermatology and have focused on other domains, such as enterprise knowledge management and more general AI versatility. Importantly, the comparatively higher number of studies examining GPT-based models reinforces their established role in dermatology research while raising the opportunity for additional explorative work around their diagnostic potential.
